# Cytological Observation of Distant Hybridization Barrier and Preliminary Investigation of Hybrid Offspring in Tea Plants

**DOI:** 10.3390/plants14132061

**Published:** 2025-07-05

**Authors:** Xiaoli Mo, Yihao Wang, Yahui Huang, Zhen Zeng, Changyu Yan

**Affiliations:** College of Horticulture, South China Agricultural University, Guangzhou 510642, China; mxiaol1997@163.com (X.M.); wangyhtea@163.com (Y.W.); 13501513191@163.com (Y.H.)

**Keywords:** distant hybridization, reproductive barrier, hybrid offspring

## Abstract

The undertaking of distant hybridization holds paramount significance for the innovation of tea germplasm resources and the cultivation of superior, specialized tea varieties. However, challenges manifest during the process of tea plant distant hybridization breeding, with reproductive barriers impeding the successful acquisition of hybrid progeny; the precise stages at which these barriers occur remain unclear. In this study, utilizing *Camellia sinensis* cv. Jinxuan as the maternal parent, as well as *C. gymnogyna* Chang and *C. sinensis* cv. Yinghong No.9 as the paternal parents, interspecific distant hybridization (DH) and intraspecific hybridization (IH) were conducted. The investigation involved the observation of pollen germination and pollen tube behavior on the stigma, the scrutiny of the developmental dynamics of the ovary post-hybridization, and the examination of the stages and reasons for reproductive disorders during tea tree distant hybridization. The findings indicate that both IH and DH exhibit pre-fertilization barriers. The pre-embryonic development of hybrids obtained from DH is normal, but there is a significant fruit drop during the stage of fruit development. The germination rate of mature seeds obtained from DH is low, and there are pronounced post-fertilization disorders, which are the primary reasons for the difficulty in achieving successful tea plant distant hybridization. An analysis of the genetic variation in phenotypes and chemical components in the progeny after distant hybridization revealed widespread variation and rich genetic diversity. The identification of progeny with a high amino acid and caffeine content holds promise for future production and breeding, providing valuable theoretical references for the selection of parents in the creation of low-caffeine-content tea germplasm resources.

## 1. Introduction

The tea plant (*Camellia sinensis*) is a widely cultivated and economically significant crop. Tea, processed from fresh tea plant leaves, has been shown to possess health benefits such as antioxidant, antibacterial, and anti-inflammatory properties, as well as blood glucose and lipid-lowering effects [1,2,3,4,5], making it highly favored by consumers. China boasts a rich variety of tea categories, expansive tea-growing regions, and abundant tea plant germplasm resources. Wild tea tree germplasm resources, including *C. tachangensis* Zhang, *C. crassicolumna*, *C. gymnogyna* Chang, and *C. ptilophylla* Chang, have been discovered in various regions [6,7,8,9,10]. Despite these wild germplasm resources containing diverse and unique functional components, the processed tea derived from them often exhibits a poor quality [11].

To meet evolving market demands and address biological pressures, the genetic improvement of the tea plant has become crucial. Currently, conventional breeding methods, encompassing selection breeding, introduction, and hybrid breeding [11], dominate genetic improvement efforts for tea plants. Hybrid breeding involves mating or combining parents with different genetic compositions to produce hybrid offspring, which are then selectively cultivated into new varieties. Although some new varieties have been obtained through intraspecific hybridization [12,13,14,15,16], none yet meet the societal demand for high efficiency and quality offspring with desirable traits, including good resistance. Wild germplasm resources, such as those from *C. gymnogyna* Chang, are valuable breeding materials and provide the material foundation for conducting hybrid breeding work. The rational utilization of these resources is essential for improving and innovating tea plant traits [17].

Distant hybridization, which involves interbreeding between species, genera, and individuals with distant genetic relationships, combines the traits of genetically distinct individuals. It breaks interspecific boundaries, significantly increases genetic variation, and serves as a crucial avenue for germplasm innovation and variety improvement [18]. Through distant hybridization, superior traits from different species can be combined to create new varieties with desired characteristics, thereby enhancing the economic benefits of tea production. Liu et al. first attempted the distant hybridization of tea plants with *C. taliensis* and *C. sinensis* cv. Fuding Dabaicha. However, due to various reasons, the germination rate of the seeds was zero. They managed to obtain some F1 seedlings using embryo rescue technology [19]. Wang et al. conducted distant hybridization using *C. ptilophylla* Chang, Hongyacha, and high-quality cultivated varieties, obtaining a small number of offspring. However, the F1 generation exhibited poor adaptability and growth potential [20]. Distant hybridization holds broad application potential but is accompanied by challenges, such as severe incompatibility and low seed setting rates, which constrain the application of distant hybridization in the Sect. Thea [21,22,23]. Therefore, understanding the reasons and stages of reproductive barriers during the process of distant hybridization in tea trees is crucial for obtaining interspecific distant hybridization offspring within Sect. Thea.

*Camellia sinensis* and *C. gymnogyna* are two distinct species within the *Camellia Sect. Thea*. Research indicates that wild tea plants produce catechins with simpler structures and lower levels of esterification compared to the more complex catechins accumulated in domesticated tea plants following long-term cultivation. Studies have shown that *C. sinensis* accumulates higher levels of esterified catechins, while *C. gymnogyna* exhibits a less complex catechin profile. Additionally, genetic analyses confirm that these two species are distantly related [24,25]. In this study, hybridization experiments were conducted using *Camellia sinensis* cv. Jinxuan (JX) as the maternal parent, with *C. gymnogyna* Chang (CGC) and *C. sinensis* cv. Yinghong No.9 (YH) as the paternal parents. Through an observation of pollen germination, pollen tube growth, and early embryo development after pollination, the fruit setting rate, fruit drop rate, and seed germination rate post-hybridization were statistically analyzed to determine the main obstacles in distant hybridization. This lays the foundation for the realization of distant hybridization in the tea subgenus. Simultaneously, the study explores the differences between interspecific and intraspecific hybridization in tea plants, aiming to elucidate the reasons for reproductive barriers during the distant hybridization process between JX and CGC. The research provides a theoretical basis for interspecific distant hybridization in *Camellia Sect. Thea* and statistically analyzes the genetic variation in phenotypes and major biochemical components of distant hybridization offspring. This offers theoretical support for the early selection of hybrid offspring and the further utilization of interspecific hybridization advantages, while also providing new and excellent materials for tea plant breeding.

## 2. Results

### 2.1. Post-Pollination Pollen Germination and Tube Growth in Female Stigma

The TTC staining method was employed to rapidly assess pollen viability. This facilitated a swift evaluation of the vitality of paternal pollen before hybrid breeding, enabling the selection of tea tree pollen with a higher vitality, therefore enhancing the efficiency of artificial hybridization. The observed pollen viability for CGC and YJ was approximately 80%, rendering them suitable for pollination (Figure S1).

**Figure 1:**
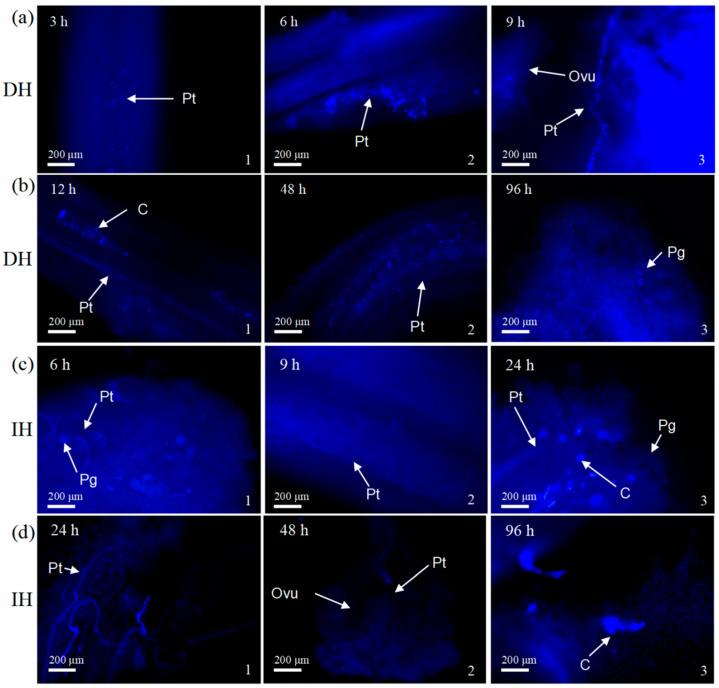
Growth of pollen tubes in the pistil after artificial pollination in two hybrid combinations. (**a**) Growth of pollen tubes after the style reaches the base in distant hybridization (DH). Three hours post-pollination, some pollen tubes (Pts) can already be seen penetrating the style interior. By 6 h, the pollen tubes had grown to the middle of the style. At 9 h, some pollen tubes were seen to twist and grow into the ovary, uniting with the ovules (Ovus). (**b**) Growth of pollen tubes after the style reaches the base in distant hybridization. By 12 h, increased pollen germination and callose (C) production were observed on the stigma. At 48 h, pollen tubes (Pts) entered the ovary with spiral growth and intermittent callose accumulations near the ovules. At 96 h, pollen grains (Pgs) were present on the maternal stigma, with some showing signs of shrinkage. (**c**) Growth of pollen tubes before the style reaches the base in intraspecific hybridization (IH). At 6 h post-pollination, a small number of pollen grains (Pgs) were observed on the stigma’s surface, with some pollen tubes (Pts) just penetrating through the papilla cell layer into the interior of the stigma. By 9 h, pollen tubes had grown to the middle of the style. At 12 h, the number of germinating pollen grains had increased, and a significant amount of callose (C) was produced at the two-thirds point along the style. (**d**) Growth of pollen tubes after the style reaches the base in intraspecific hybridization. By 24 h post-pollination, pollen tubes (Pts) had reached the base of the style, but exhibited twisting and coiling, along with callose (C) accumulation, which prevented normal entry into the ovary. Contact between pollen tubes and ovules (Ovus) was not observed until 48 h, and it was not until 96 h that callose (C) phenomena at the embryo sac indicated the completion of fertilization.

Using JX as the female parent, interspecific distant hybridization (DH) and intraspecific hybridization (IH) were conducted with two male parents—CGC and YH. The staining of the pistil tissues at 3 h, 6 h, 9 h, 12 h, 24 h, 48 h, 72 h, 96 h, and 120 h post-pollination with aniline blue solution revealed notable differences in the growth rates of pollen tubes within the styles. Notably, in the DH group, pollen tube growth was observed at as early as 3 h post-pollination, with pollen tubes reaching a certain length by 9 h post-pollination, visible at the base of the style (Figure 1a). In contrast, the IH group exhibited a delayed pollen tube emergence, with tube growth being observed on the stigma at 6 h post-pollination, reaching the middle of the style at 9 h, and finally extending to the base of the style by 24 h post-pollination (Figure 1c).

However, after reaching the base, the growth of pollen tubes in the DH group was slower than that in the IH group. Both the IH and DH groups experienced varying degrees of impediment to pollen tube growth after reaching the base. In the IH group, 24 h post-pollination, distorted and entangled pollen tubes, along with the accumulation of callose, were observed, hindering normal entry into the ovary. The phenomenon of pollen tubes contacting the ovule was only observed at 48 h post-pollination, with the maternal stigma showing no pollen adhesion at 96 h, accompanied by a noticeable callose blockage within the ovary, indicative of completed fertilization (Figure 1d). In the DH group, numerous callose blockages were present at different locations during pollen tube germination. Despite hindrances, a substantial number of pollen tubes in the DH group grew normally and rapidly reached the base. However, upon reaching the base, an intensified callose reaction was noted during pollen tube germination and growth, resulting in numerous intermittent callose blockages throughout the style. After entering the ovary, a considerable obstruction of callose hindered the union with the ovule. At 96 h post-pollination, the maternal stigma still retained pollen grains, some of which had shrunk; additionally, some pollen tubes reached the ovary tissues and interacted with the ovules successfully, as evidenced by callose reactions within the ovule and the enlargement of the embryo sac (Figure 1b).

Overall, the pollen tubes of the DH group grew faster, but their growth was hindered after reaching the base of the style, making it difficult for the pollen tubes to unite with the ovules. In IH, the pollen tubes grew relatively slowly but were eventually able to complete the fertilization process. These results reveal the different biological characteristics and possible hybridization barriers between distant and intraspecific crosses during pollination, providing important information for understanding of the hybridization mechanism of tea plants.

### 2.2. Early Embryonic Development After Pollination in the DH Group

To further elucidate the causes of cross-incompatibility in the distant hybridization of tea plants, we conducted a micro-observation of early embryonic tissue sections from the DH group at intervals ranging from 0 h to 30 days post-pollination.

**Figure 2:**
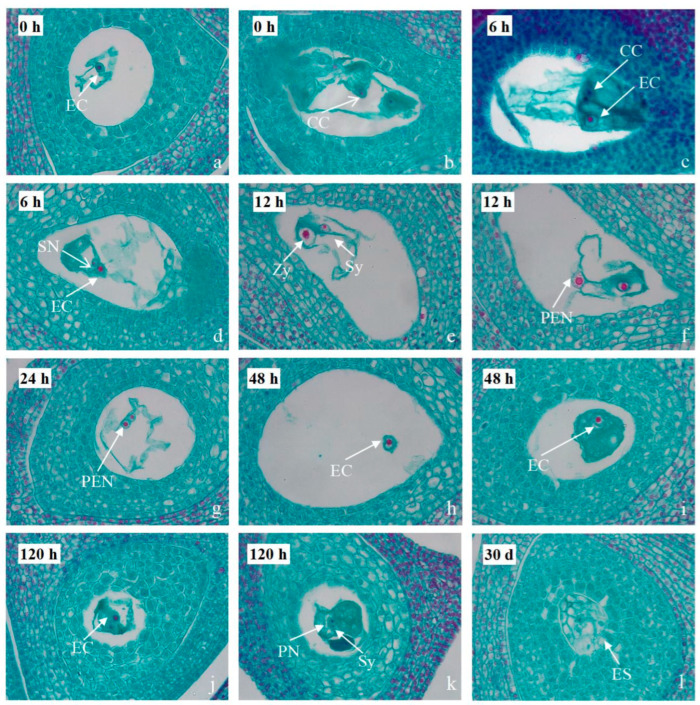
Early embryonic development in distant hybridization (DH) crosses using *C. gymnogyna* (CGC) as the pollen donor. (**a**–**l**) Longitudinal sections of embryo sacs from JX (♀) × *CGC* (♂) hybrids at 0 h to 30 days post-pollination (DPP). (**a**,**b**) 0 h DPP: Mature female gametophyte showing egg cell (EC) and central cell (CC). (**c**,**d**) 6 h DPP: Pollen tube entry; sperm nucleus (SN) migrating toward egg cell. (**e**,**f**) 12 h DPP: Syngamy initiation with degenerated synergids (Sys); primary endosperm nucleus (PEN) formation. (**g**) 24 h DPP: Distinct pen alongside unfertilized polar nucleus (PN). (**h**,**i**) 48 h DPP: Sperm chromatin adhering to egg nuclear membrane. (**j**,**k**) 120 h DPP: Karyogamy completion in zygote (Zy); sperm nucleus fusing with polar nucleus. (**l**) 30 d DPP: Developing cellularized endosperm (ES).

Microscopic observations of early embryonic tissue slices were conducted from 0 h to 30 days post-pollination in the DH group, with CGC as the paternal parent. At 0 h post-pollination, clear observations of the egg cell (Figure 2a) and central cell (Figure 2b) in the center of the embryo sac indicated the maturity of the pistil’s ovule. At 6 h post-pollination, the structural features of the follicle were visible (Figure 2c), and the sperm nucleus was preparing to fuse with the egg cell (Figure 2d). By 12 h post-pollination, the fusion of the sperm nucleus and egg nucleus was observed, accompanied by the degeneration of the synergids (Figure 2e). The fusion of the sperm nucleus with the polar nuclei formed the primary endosperm nucleus (Figure 2f). This observation aligns with the fluorescence results of the pollen tube, which, after 9 h post-pollination, exhibited twisted growth, entering the ovary and interacting with the ovule. At 24 h post-pollination, the formation of the primary endosperm nucleus was evident, with another unfertilized polar nucleus positioned alongside (Figure 2g). By 48 h post-pollination, the sperm nucleus adhered to the egg nucleus membrane or entered the egg cell, with dispersed chromatin (Figure 2h,i). At 120 h post-pollination, the fusion of the egg and sperm nuclei was observed, with the sperm nucleus entering one of the polar nuclei (Figure 2j,k). At 15 days post-pollination, the observation of the developing primary endosperm nucleus in the embryo sac, with the primary endosperm nucleus located at the center of the embryo sac, was consistent with the fluorescence observation after 120 h of pollination. At 30 days post-pollination, the development of the endosperm was observed (Figure 2l). These findings indicate that the endosperm is still developing at 120 h~30 d after pollination in the DH group.

### 2.3. Observation of Hybrid Fruiting Development and Seed Germination

**Figure 3:**
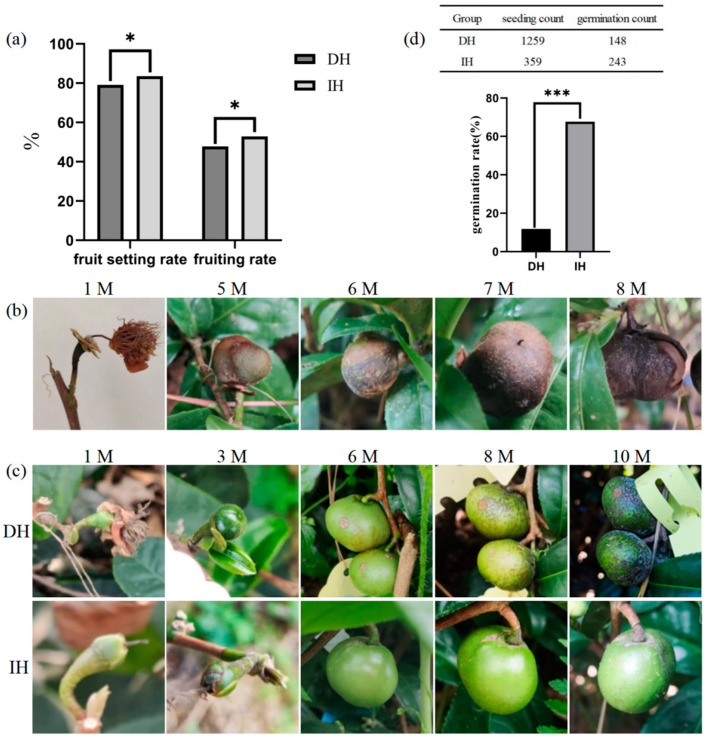
Observational results of fruit development. (**a**) Fruit setting rate and fruiting rate of two hybrid combinations ten months after pollination. (**b**) Manifestations of abnormal fruit development after pollination. (**c**) Differences in the development of normal fruits between the two hybrid combinations after pollination. DH: distant hybridization; IH: intraspecific hybridization. (**d**) Comparison of the number of seeds sown and the number of germinations in the two hybrid combinations. Note: * Correlation is significant at the 0.05 level; *** correlation is highly significant at the 0.001 level.

In this study, 1023 pollinations were conducted in the DH group, and 242 pollinations were conducted in the IH group. Continuous observations were made after pollination, and the resulting fruits were harvested ten months after pollination. A statistical analysis revealed that the fruit-setting rate and fruiting rate in the DH group were significantly lower than those in the IH group. The fruit-setting rate in the DH group was 79.1%, with a fruiting rate of 47.8%, while the IH group exhibited a fruit-setting rate of 83.5% and a fruiting rate of 52.9% (Figure 3a).

Monthly observations of fruit development in the two hybrid combinations revealed various conditions in the ovaries, leading to abortion and fruit drop at different stages of pollination. One month after pollination, some ovaries exhibited signs of atrophy and abortion, with the flower stalk changing from green to red but not detaching. Even if some ovaries developed into young fruits, a portion failed to grow normally until maturity. Between 5 and 10 months post-pollination, underdeveloped fruits lost their luster, changed color from green to brown, and showed varying degrees of depression or even detachment. The volume of these abnormal fruits was only equivalent to the size of the ovaries that developed normally 2–3 months after pollination. Internally, the seeds formed hollow cavities, and the cotyledons were slightly moist and flattened (Figure 3b). Observations indicated that the fruit drop rate in both the DH and IH groups was highest at one month after pollination. While the IH group showed no fruit drop after 7 months post-pollination, the DH group experienced continuous fruit drop from pollination to harvest (Table S1).

For normally developing hybrid fruits, three months after pollination, the DH group exhibited larger young fruit volumes compared to the IH group. During the fruit development process, the DH group displayed a more noticeable color change in the fruit peel, transitioning from green to yellow–green and eventually to yellow–brown or brown. In the same period, the IH group exhibited fewer color changes, maintaining a green or light-green color for a longer duration. Ten months after pollination, the DH group showed mature fruits, while no such fruits were observed in the IH group, indicating faster fruit development in the DH group (Figure 3c).

A statistical analysis of seed germination from the two hybrid combinations revealed that the germination rate of distant hybridization seeds was significantly lower than that of intraspecific hybridization. The DH group exhibited a germination rate of only 11.8%, while the IH group had a high germination rate of 67.7% (Figure 3d).

### 2.4. Analysis of Phenotypic Traits in Distant Hybrid Offspring

As shown in the previous study, although the pollen tubes of the DH group encountered obstacles to growth at the base of the style, a considerable number of pollen tubes eventually successfully united with the ovules, completed fertilization, and some seeds were successfully harvested and sown. In order to understand the effect of successful interspecific pollination on the growth and genetic diversity of the progeny, we investigated the progeny of the obtained crosses to gain a more comprehensive understanding of the effect of distant hybridization on the genetic diversity of tea plants.

**Figure 4:**
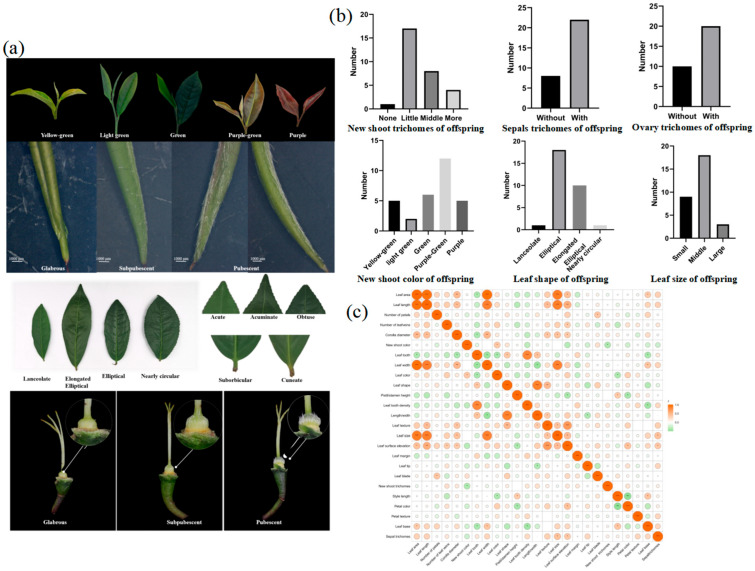
Phenotypic observations of the distant hybrid offspring. (**a**) Some phenotypic traits of new shoots, mature leaves, and flowers in the distant hybrid offspring. (**b**) Types and quantities of new shoot trichomes, sepal trichomes, ovary trichomes, new shoot color, mature leaf shape, and leaf size in the distant hybrid offspring. (**c**) Results of phenotypic trait correlation analysis in hybrid offspring. Note: * Correlation is significant at the 0.05 level; ** correlation is highly significant at the 0.01 level; *** correlation is highly significant at the 0.001 level.

An investigation was conducted on 30 distant hybrid offspring with relatively uniform growth traits, and the results are presented in the Supplementary Table (Table S2). The yield of young tea plant per plant was measured using the weight of pruning materials [26]. The average plant height of the hybrid offspring was 111.63 cm, the average basic stem diameter was 1.87 cm, and the average pruning material weight was 235.07 g, indicating a high production potential. Significant morphological differences were observed in the hybrid offspring resulting from the cross between JX and CGC, showing various degrees of genetic variation and widespread trait segregation (Figure 4a).

A statistical analysis revealed that the coefficient of variation for 20 descriptive traits ranged from 16.33% to 71.92%, with the three highest coefficients related to trichome traits—ovary trichomes (71.92%), sepal trichomes (61.33%), and new shoot trichomes (51.79%) (Table S3). The new shoots of JX had short and dense trichomes, while CGC had no trichomes on new shoots or ovaries. The hybrid offspring exhibited four types of new shoot trichomes, with the majority being intermediate. The proportion of offspring with fewer new shoot trichomes was 56.67%, and those without trichomes accounted for only 3.33%. Approximately one-third of the hybrid offspring had ovaries without trichomes. The maternal parent JX had a yellow–green color for bud leaves, while the paternal parent CGC had a light-green color with a hint of purple. The hybrid offspring exhibited five colors for bud leaves, including purple, purple–green, green, light-green, and yellow–green, with purple–green being the most predominant (Figure 4b).

Considerable separation was observed in mature leaf traits, especially leaf shape. JX’s mature leaves were elliptical, while CGC’s were elongated elliptical. The hybrid offspring displayed four leaf shapes—lanceolate, elongated elliptical, elliptical, and nearly circular. Approximately 60% were consistent with the maternal parent, being elliptical, while one-third matched the paternal parent, being elongated elliptical. Lanceolate and nearly circular shapes each accounted for only 3.33%.

A correlation analysis of phenotypic traits in the hybrid offspring revealed that as bud leaf color deepened, bud leaf trichomes decreased. Wider leaves tended to have a more circular base, and a larger length-to-width ratio led to a sharper leaf tip. Longer styles were associated with a higher probability of the pistil being higher than the stamen. In addition to correlations between various traits in different organs, there were close connections between bud leaves, mature leaves, and flowers. Offspring with larger leaves tended to have larger flowers, and those with more bud leaf trichomes also had more ovary trichomes (Figure 4c).

The performance of hybrid offspring is shown in Tables S3 and S4, where the mid-parent value (HM) represents the difference between the F1 generation and the average of the parents, while the relative mid-parent value (RHM) represents the ratio of the mid-parent value to the parental average. The results indicate that F1 averages fall within the parental range, with no over-dominance. Out of the 27 phenotypic traits for bud leaves, mature leaves, and flowers, 11 traits showed negative mid-parent values, including bud leaf trichomes, leaf size, leaf tip, leaf texture, number of leaf serrations, corolla diameter, style length, number of petals, petal color, pistil–stamen height ratio, and ovary trichomes. Eight traits exhibited no mid-parent advantage, and the remaining nine traits showed positive mid-parent values (Table S4).

### 2.5. Analysis of Chemical Components in Progeny of Distant Hybridization

**Figure 5:**
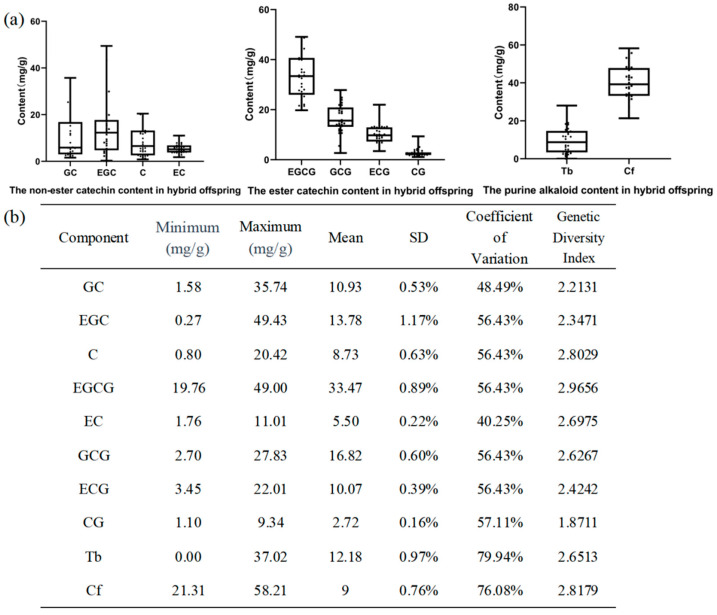
Chemical composition of the distant hybrid offspring. (**a**) Catechin and purine alkaloid fractions and contents in distant hybrid offspring. (**b**) Statistical analysis of chemical components in offspring.

The results of chemical component analysis for the parents and their hybrid progeny are presented in Figure 5a, Table S5. It is observed that the descendants with a darker leaf color exhibit a relatively higher total anthocyanin content, ranging from 0 to 0.9 mg/g. Some individuals in the F1 generation show higher levels of free amino acids, caffeine, and total anthocyanins compared to the parents, showing the phenomenon of super-parenting.

In the catechin components, except for ECG, the maximum values of the other seven monomers are significantly higher in the offspring than in the parents, and the average content of ester-type catechins in the offspring is higher than that in the parents. The parent JX has a caffeine content of 40.30 mg/g, with almost no theobromine, while the parent CGC contains only theobromine (77.08 mg/g). Unfortunately, no descendants with low or no caffeine were identified. The caffeine content in the hybrid offspring ranges from a minimum of 21.31 mg/g to a maximum of 58.21 mg/g.

There are significant differences in the composition and content of catechins between the parents and their offspring. The major catechin components in JX are, in descending order of content, as follows: EGCG > EGC > C > ECG > GCG > GC > EC > CG. In CGC, the order is ECG > EGCG > GCG > EC > CG > EGC > C > GC. The hybrid offspring population shows substantial variation in the content of different catechin substances, with the average catechin content in descending order being observed as follows: EGCG > GCG > EGC > GC > ECG > C > EC > CG. Except for CG, the Shannon–Wiener diversity index for other chemical components is greater than 2, with the highest value being observed for EGCG (2.9656), followed by ester-type catechins and caffeine at 2.8242 and 2.8179, respectively. This indicates that the chemical components in hybrid offspring have a rich diversity (Figure 5b).

## 3. Discussion

### 3.1. Phenomena of Abnormal Pollen Tube Growth in Interspecific and Intraspecific Hybridization Offspring

Tea plants are self-incompatible, and their mechanism of incompatibility has been extensively studied [27,28,29,30,31]. In recent years, significant progress has been made in the hybridization of Sect. Thea, but most studies have focused on the genetic traits and quality differences in hybrid offspring, with limited reports on changes in pollen tube growth and embryo development after pollination. Distant hybridization is an effective method for broadening the genetic foundation of cultivated varieties and creating genetic resources. However, it is often challenging due to obstacles such as different genetic information between species and incompatible developmental processes, which make successful distant hybridization difficult. Failure to complete fertilization and form a zygote is generally considered a primary factor leading to the failure of distant hybridization [32,33,34].

In this study, using JX as the female parent, interspecific distant hybridization (DH) and intraspecific hybridization (IH) were conducted with two male parents—CGC and YH. The growth status of pollen tubes and the development of embryos after pollination were observed for both combinations. The results show that before the pollen tube extends to the base, the pollen tube germination rate in the DH group is faster than in the IH group. Pollen tubes in the DH group germinate at as early as 3 h after pollination; at 9 h, pollen tubes can be observed at the base of the style. In contrast, in the IH group, pollen tube germination is observed 6 h after pollination, and it takes 24 h to extend to the base of the style. This difference may be due to variations in the efficiency of pollen germination between the YH and CGC varieties. However, after the pollen tube reaches the base of the style, both the DH and IH groups exhibit the twisting and entwining of the pollen tubes at the base of the style, and there is a significant accumulation of callose in the style. This phenomenon is one of the manifestations of pre-fertilization barriers [35]. Callose is a β-1,3-glucan-linked polysaccharide that plays an important regulatory role in plant sieve tube metabolism and gametophyte development. However, the excessive accumulation of callose can hinder pollen tube elongation, preventing the pollen tube from reaching the ovary to complete fertilization, thereby affecting fruiting [36,37,38]. Despite this, most pollen tubes from both combinations can still germinate normally and grow rapidly in the style, although the growth of pollen tubes entering the ovary is slow. Eventually, a small number of pollen tubes reach the ovule and continue with double fertilization. Both interspecific and intraspecific hybridization show a certain pre-fertilization barrier, but this does not seem to be the main factor affecting the difference in fertilization and fruiting between the two.

The dynamic observation of embryo development after fertilization did not reveal embryo abortion 30 d after pollination in this experiment, indicating that the early development of hybrid embryos from the distant hybridization of DH is normal. Observations of hybrid fruit development and seed germination indicate that the fruit-setting rate of both hybrid combinations is above 79%, and the fruiting rate is close to 50%. However, the DH group exhibits a certain degree of fruit drop during the growth and development process, with a lower germination rate and a more severe phenomenon of the non-viability of hybrids. Hybrid non-viability refers to the situation where the zygote can survive, but embryonic death occurs at the embryonic development stage or the individual does not develop to sexual maturity, thus not producing offspring. Many factors can cause non-viability, and the main reasons are the lack of coordination in genotypes and the failure of growth regulation [39,40]. The post-fertilization barrier caused by interspecies relationships manifests as a decrease in fruit-setting rate and even affects the survival rate of offspring [41]. Through this study, it is evident that post-fertilization barriers are the main reason for obtaining fewer offspring in distant hybridization.

The results of this study indicate that distant hybridization has a certain degree of affinity, and embryos can develop normally in the early stages after successful fertilization. However, severe fruit drop occurs in the later stages, leading to the failure of hybridization. Therefore, to obtain more distant hybrid offspring, it is necessary to collect embryos with normal early development for embryo rescue.

### 3.2. Analysis of Phenotypic Genetic Variation in Distant Hybrid Offspring

Tea plants have large genomes and a high heterozygosity, leading to the extensive segregation of traits in interspecific distant hybrid offspring. In this study, the level of variation in hybrid offspring was high, with a coefficient of variation for bud and leaf phenotypes ranging from 27.53% to 51.79%, for mature leaves from 9.92% to 47.75%, and for flower phenotypes from 11.19% to 71.92%, indicating a rich genetic diversity.

Previous studies on the genetic tendencies of tea leaf traits have shown that traits such as new shoot trichomes tend to favor the maternal parent [42,43]. In this study, the paternal parent used was CGC, which has no trichomes on new shoots and ovaries, and its mature leaves are elongated elliptical. The maternal parent, JX, has short and dense trichomes, with elliptical mature leaves. The hybrid offspring generally inherited the density of new shoots and ovaries from the maternal parent, with only one individual having no trichomes on new shoots. About one-third of the offspring had ovaries without trichomes. The mature leaves of the offspring were mostly elliptical, indicating the maternal inheritance of new shoot trichomes, ovary trichomes, and leaf shape, consistent with previous research results [42,43,44,45]. Leaves are important economic organs of tea, and their phenotypic traits are influenced by both genetic and environmental factors, with some traits being more affected by the environment [46]. In this study, both parents had flat leaf surfaces, wedge-shaped bases, and wavy leaf margins. However, the hybrid offspring did not always exhibit these traits, which is similar to the results found in other woody plants [47,48].

Genetic studies on leaf color in tea plants have mainly focused on albino varieties, with little research on purple-leaf varieties. One study suggests that new shoot color in the “Baijiguan” variety is controlled by multiple genes and is a quantitative trait [49]. In this study, the parental leaf colors were light-green with purple, or were yellow–green, while most hybrid offspring showed various shades of purple and deep purple, indicating that new shoot color in tea plants is controlled by multiple genes, and purple is a dominant trait over green, which is consistent with studies on leaf color in lemons and other plants [49,50].

Tea plants have highly heterozygous genotypes, and traits in hybrid offspring exhibit extensive segregation. Studying their genetic patterns is challenging, but through a statistical analysis of the phenotypic expressions in hybrid offspring, we can identify the genetic tendencies of various traits. Summarizing the genetic tendencies of offspring traits in different hybrid combinations can help us select more suitable hybrid combinations and improve breeding efficiency.

### 3.3. Analysis of Genetic Variation in Biochemical Components of Distant Hybrid Offspring

Biochemical components are essential foundations for the quality attributes such as color, aroma, and taste of tea. Hybridization is an effective means to create new varieties, and it holds the potential to obtain tea plant varieties with specific chemical compositions. Amino acids are the main source of the fresh and brisk taste of tea leaves, and the general content of free amino acids in tea ranges from 1% to 4%. Epigallocatechin gallate (EGCG) is the most abundant active substance in tea polyphenols, known for its antioxidant properties. Caffeine has a stimulating effect on the central nervous system. Varieties with specific amino acid, EGCG, and caffeine contents have long been breeding goals in tea research. For instance, a tea variety with an EGCG content higher than 13% is considered a high-EGCG resource, while a caffeine content lower than 1.5% is regarded as a low-caffeine resource, and a caffeine content higher than 5% is considered as a high-caffeine resource [51,52,53].

The biochemical components of the hybrid offspring were analyzed, revealing that three individuals had a total free amino acid content exceeding 4.8%, and one individual exceeded 5%. The EGCG content ranged from 1.98% to 4.91%, and no hybrid offspring were found with a high EGCG content. There were five individuals identified as high-caffeine resources. No hybrid offspring with a low caffeine content were observed, and the lowest caffeine content in the offspring was 2.13%, which is significantly lower than that of the maternal parent. This suggests that by backcrossing the hybrid offspring with a low caffeine content to the paternal parent, it may be possible to obtain hybrid offspring with a caffeine content lower than 1%. Studies by Yang, Zhong, and others have identified multiple caffeine synthase allele genes, and the results indicate that the *TCS1e* gene is of significant importance for breeding low-caffeine tea plants. The subsequent identification and utilization of the *TCS1* gene in the low-caffeine hybrid offspring and CGC used in this study may be considered [54,55].

The analysis of genetic variation in biochemical components of hybrid offspring provides preliminary insights into the genetic characteristics of tea leaf biochemical components. It offers guidance for the selection of hybrid parents. The variation coefficients and genetic diversity of biochemical components in hybrid offspring indicate significant genetic diversity, with variation coefficients ranging from 7.60% to 76.08%, with caffeine showing the highest variation. The variation range for catechin components is 26.63% to 72.65%, with C showing the highest variation coefficient and EGCG showing the lowest, which is consistent with previous research results [56,57].

## 4. Materials and Methods

### 4.1. Plant Materials and Artificial Pollination

This experiment involved the design of two hybrid combinations, both with *Camellia sinensis* cv. Jinxuan (JX) as the maternal parent and either *C. gymnogyna* Chang (CGC) or *C. sinensis* cv. Yinghong No.9 (YH) as the paternal parent. The combination JX × YH represents intraspecific hybridization (IH), while JX × CGC represents distant hybridization (DH). The plant materials are preserved at SCAU Teaching and Research Base in Zengcheng District, Guangzhou.

Pollen was collected during the balloon stage of flower development. Flower buds in the balloon stage were collected, spread in a cool and dry place, and after 24 h when the pollen matured, it was tapped out, purified, and then collected in a 50 mm culture dish. The pollen vitality was assessed before pollination [58].

On a clear and windless morning, branches with flowers from the lower part of JX were selected. Well-developed buds with unopened petals were chosen, and after the careful removal of stamens, pollination was performed, followed by bagging. Female reproductive organs (pistils) were collected at different time points after pollination (3 h, 6 h, 9 h, 12 h, 24 h, 48 h, 72 h, 96 h, and 120 h for pollen germination observation; 0 h, 6 h, 12 h, 24 h, 48 h, 120 h, 15 d, and 30 d for early embryonic development after pollination). Approximately 10 pistils were randomly selected at each time point, fixed with FAA solution(AR grade), and stored in a refrigerator at 4 °C for at least 24 h.

### 4.2. Observations on Pollen Germination and Early Embryo Development

The materials fixed for more than 24 h were taken out from FAA, rehydrated for 30 min, soaked in distilled water for 30 min, longitudinally cut along the style with a blade, and the halves of the pistils were placed in a 2.5 mL centrifuge tube. About 2/3 of 10% anhydrous sodium sulfite solution was added to the centrifuge tube, and it was softened in a 95 °C water bath. After softening, the material was washed with distilled water for 30 min, before being stained with 0.1% water-soluble benzidine solution in the dark for 30 min. The stained pistils were placed on glass slides with glycerol droplets for even spreading, and observations were made using a Zeiss fluorescence microscope (DH1810, Carl Zeiss (Shanghai, China) Management Co., Ltd).

The fixed materials were dehydrated, embedded in paraffin, and sectioned with a thickness of 8–10 μm. The sections were stained with safranin and fast green, and neutral gum was used for sealing. Observations were made using a Zeiss fluorescence microscope.

All drugs, with the exception of FAA fixative, which was purchased from Labgic Technology Co., Ltd. (Beijing, China), were obtained from Macklin Biotechnological Co., Ltd. (Shanghai, China).

### 4.3. Statistics on Fruiting and Seed Germination Rates

One month after pollination, regular observations were conducted either every 15 days or intermittently for the two hybrid combinations. The status of fruit setting and development was recorded, and both the fruit-setting rate and the fruit-drop rate were calculated. This observation continued until approximately one week before and after the first frost of the following year when tea fruits were harvested; then, the fruiting rate was assessed.

Fruit setting rate = number of fruit set/number of pollination × 100%.

Fruiting rate = number of fruits/number of pollination × 100%.

After harvesting the fruits, they were thinly spread in a well-ventilated and dry area to facilitate the dehydration and cracking of the fruit shells. Seeds were extracted, soaked in water for 2 days, and seeds that sank to the bottom were selected as planting materials. Simultaneously, the number of seeds was counted, and appropriate group labels were applied. After planting, the seed germination rate was recorded three months later.

Seed germination rate = number of germination/number of pollination × 100%.

### 4.4. Phenotypic Investigation and Biochemical Component Analysis of Distant Hybrid Offspring

A phenotypic investigation and a biochemical component determination of the hybrid offspring were conducted following the methodology outlined by Chen [59]. One bud with two leaves was collected from both parents and hybrid offspring, fixed using a microwave, dried, ground to pass through a 60-mesh sieve, and utilized for the determination of biochemical components. The quantification of catechins and purine alkaloids was performed using the HPLC method established by our laboratory [9], and the total amount of free amino acids was determined in accordance with the Chinese national standard.

### 4.5. Data Analysis

The genetic variability and diversity of the F1 generation was assessed through the utilization of the coefficient of variation (CV) and the Shannon–Wiener genetic diversity index (H’), respectively. IBM SPSS Statistics 26 was employed for a differential analysis and correlation analysis of the collected data.

## 5. Conclusions

This study suggests that distant hybrid offspring of Sect. Thea exhibit a high genetic diversity in biochemical components. The identification of specific offspring with a high amino acid and high caffeine content may have value in future production and breeding efforts. Additionally, this study provides theoretical references for the selection of parental resources in the creation of low-caffeine tea tree germplasm.

## Data Availability

All relevant data and figures in this study can be found within the article and its Supporting Materials.

## Supplementary Materials

The following supporting information can be downloaded at https://www.mdpi.com/article/10.3390/plants14132061/s1. Figure S1: TTC staining method for culturing pollen from the male parent; Table S1: Comparison of the number of fruit abscissions at the same time in two groups of hybrid combinations; Table S2: Phenotypic traits of parents and offspring; Table S3: Statistical analysis of morphology in hybrid offspring; Table S4: Advantageous performance of hybrid progeny; Table S5: Chemical composition of parents and offspring. Table S6: Descriptive trait assignment table.
