# Supplementary figures and images for: Cytological Observation of Distant Hybridization Barrier and Preliminary Investigation of Hybrid Offspring in Tea Plants
Source: Plants (Basel). 2025 Jul 5;14(13):2061. doi: 10.3390/plants14132061 (PMC12251903; doi:10.3390/plants14132061)

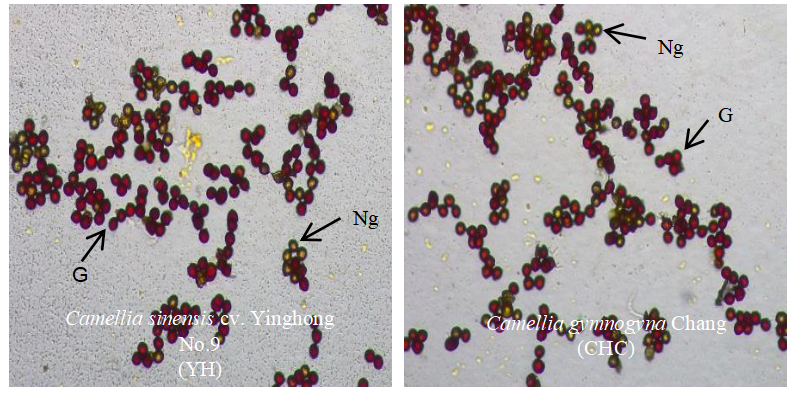

Supplement: Supplementary file 1 [file plants-14-02061-s001.zip › fig S1.tif]
